# HMGA2 induces epithelial-to-mesenchymal transition in human hepatocellular carcinoma cells

**DOI:** 10.3892/ol.2013.1193

**Published:** 2013-02-15

**Authors:** YIZHOU LUO, WENFENG LI, HUI LIAO

**Affiliations:** 1 Department of Oncology and Hematology, Jun Xie Hospital, Nanjing, Jiangsu 210002;; 2 Department of Radiation Oncology, First Affiliated Hospital of Wenzhou Medical College, Wenzhou, Zhejiang 325000, P.R. China

**Keywords:** epithelial-to-mesenchymal transition, human high-mobility group A2, hepatocellular carcinoma, Snail, Twist

## Abstract

Epithelial-to-mesenchymal transition (EMT) is an important event during tumorigenesis. The human high-mobility group A2 (HMGA2) is a chromatin-binding protein, which contains three AT-hook domains that enable its binding to the minor groove of DNA. HMGA2 organizes protein complexes on enhancers of various genes to regulate gene expression and cell differentiation. The HMGA2 protein has been reported to be overexpressed in many types of cancer. It is not known, however, whether HMGA2 regulates EMT in human hepatocellular carcinoma (HCC) cell lines, and the mechanism(s) have not been fully elucidated. In this study, the expression of HMGA2 in five HCC cell lines was examined. The levels of HMGA2 expression among the five HCC cell lines coincided with their invasiveness. The variation in HMGA2 expression significantly correlated with the expression of several putative EMT markers. In addition, assessment of the invasive potential, following transfection with HMGA2-siRNA, demonstrated that the rate of cell migration was significantly reduced, suggesting that HMGA2 may be an important contributor to the invasion of tumor cells and that expression levels of HMGA2 influence the metastatic behavior of HCC cells. To further confirm the conclusion and explore the molecular mechanism through which HMGA2 induces EMT, we found that HMGA2 upregulates the expression of Twist and Snail in HCC cell lines. In conclusion, this present study is the first to show that HMGA2 effectively regulates EMT to induce invasion and metastasis in HCC cells. The function of HMGA2 as an oncoprotein may be associated with several important molecules involved in invasion and metastasis of cancer cells.

## Introduction

Epithelial-to-mesenchymal transition (EMT) is an important event during tumorigenesis (1). The functional loss of E-cadherin protein, a key protein maintaining epithelial cell-cell adhesion, is a key marker in the EMT process (2). Several transcriptional repressors, such as the zinc finger factors Snail, Slug, ZEB1 and ZEB2, the basic helix-loop-helix (bHLH) factor E47 and Twist have been identified as strong repressors of E-cadherin expression and have been implicated in tumor progression (2).

Human high-mobility group A2 (HMGA2) is a chromatin-binding protein which contains three AT-hook domains that enable its binding to the minor groove of DNA. HMGA2 organizes protein complexes on enhancers of various genes to regulate gene expression and cell differentiation (3,4). HMGA2 protein is overexpressed in many types of cancer, such as lung cancer (5), ovarian cancer (6), breast cancer (7), oral squamous cell carcinoma (8), pancreatic cancer (9) and colorectal cancer (10). Previous studies have demonstrated how HMGA2 promotes cancerogenesis at the molecular level. For example, it is well-known that human telomerase reverse transcriptase (hTERT) is essential for tumor cell proliferation and self-renewal. HMGA2 increases hTERT transcription to promote tumorigenesis (11). Certain micro RNAs (miRNAs) are able to target HMGA2 gene, which inhibits tumorigenesis through the downregulation of HMGA2 protein (12). The role of HMGA2 that controls EMT has been well-described. Transforming growth factor-b (TGF-β) induces the expression of HMGA2 by activating transcription factor Smad. HMGA2 associates with Smad complexes to induce Snail and Twist expression, two established regulators of EMT, which finally leads to mesenchymal transition (13,14).

It is not known whether HMGA2 regulates EMT in human hepatocellular carcinoma (HCC) cell lines; furthermore, the mechanism(s) have not been fully elucidated.

## Material and methods

### Cell culture

PLC/PRF/5, Huh-7, HepG2, and Hep3B cells (nonmetastatic or low metastatic potential human HCC cell lines) used in this study were obtained from the American Type Culture Collection (ATCC; Rockville, MD, USA) and MHCC97-H cells human HCC cell lines with high metastatic potential were established at the Liver Cancer Institute, Zhongshan Hospital, Fudan University, Shanghai, China (15). All cells were cultured in the corresponding medium supplemented with 10% fetal bovine serum (FBS) and 1% penicillin-streptomycin and were maintained in a 37°C incubator with 5% CO_2_.

The study was approved by the Ethics Committee of Jun Xie Hospital, Nanjing, China.

### Transfection

HepG2 cells were transiently transfected with HA-tagged human HMGA2 using FuGENE HD (Roche Applied Science, Mannheim, Germany). Transient transfections of MHCC97-H cells with siRNA against human *Hmga2* (ON-TARGETplus SMARTpool L-013495; Dharmacon, Pittsburgh, PA, USA) or non-targeting siRNA control were done using DharmaFECT1 siRNA transfection reagent (Dharmacon). The effectiveness of gene overexpression or silencing was determined using real-time PCR and western blot analysis.

### Real-time PCR

Total-RNA was extracted using the RNeasy mini kit (Qiagen, Valencia, CA, USA) and the concentration detected using a biological spectrophotometer. Real-time PCR analysis was performed according to the manufacturer’s instructions (Quant SYBR-Green PCR kit, Tiangen Biotech, Beijing, China). Primers for mouse glyceraldehyde-3′-phosphate dehydrogenase (*Gapdh*) were used as a reference. The primers for *Gapdh* were 5′-TGTGTCCGTCGTGGATC TGA-3′ (sense) and 5′-CCTGCTTCACCACCTTCTTGA-3′ (antisense). The primers for *Hmga2* were 5′-TCCCTCTAA AGCAGCTCAAAA-3′ (sense) and 5′-ACTTGTTGTGGC CATTTCCT-3′ (antisense). Gene expression levels were determined with the comparative Ct method using *Gapdh* as a reference. The control condition was set to 1 or 100% and expression levels are presented as bar graphs of means ± standard error of the mean (SEM).

### Western blot analysis

A total of 1×10^7^ cells were collected. The cells were lysed and the protein concentrations were measured using a BCA Protein Assay Reagent kit (Pierce, Rockford, USA). A 20 *μ*g aliquot of the protein was subjected to 10% SDS-polyacrylamide gel electrophoresis (PAGE), and transferred to a polyvinylidene difluoride (PVDF) membrane (Bio-Rad, Hercules, CA, USA). After being blocked by incubation overnight in PBST containing 5% dry nonfat milk, the PVDF membrane was incubated with the indicated primary antibodies (1:1,000 dilution) for 2 h and incubated with a horseradish-peroxidase-conjugated secondary antibody (1:100 dilution; Proteintech, Chicago, IL, USA) for 1 h. Immunoreactive bands were visualized using an enhanced chemiluminescence (ECL) detection system (Amersham, Arlington Heights, IL, USA). β-actin was detected simultaneously as a loading control (anti-β-actin, 1:1,000 dilution; Kangchen, Beijing, China).

### Invasion and migration assay

For the invasion assay, cell culture inserts (8 *μ*m, 24-well format, Becton-Dickinson Labware, Franklin Lakes, NJ, USA) were evenly coated with diluted Matrigel (1:5 dilution with blank medium). Cells (1×10^5^) were added to the upper chamber and the lower chamber was filled with 300 *μ*l medium containing 10% FBS. The culture was maintained for 24 h. The cell migration assay was similar to the invasion assay, except that inserts were not coated with Matrigel and the culture was maintained for 24 h. Cells were fixed with 4% formaldehyde for 10 min and stained with 0.5% crystal violet for 10 min. The cells on the upper side of the filters were removed with cotton-tipped swabs. The cells on the underside of the filters were counted under a ×20 objective lens in five randomly selected fields. The results are presented as the fold change when compared with vector control cells.

### Statistical analysis

All experiments were performed in trip-licate. Results are expressed the mean ± SEM. A two-tailed Student’s t-test was performed to analyze the statistical significance of differences between experimental groups using the SPSS 11.5 software statistical package (SPSS Inc., Chicago, IL, USA). P<0.05 was considered to indicate a statistically significant result.

## Results

### Expression of HMGA2 in five HCC cell lines at the mRNA and protein level

**Figure 1:**
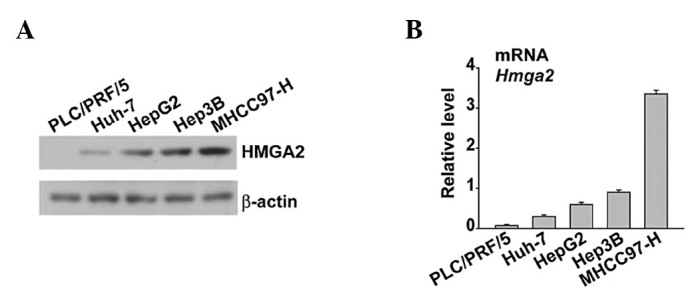
The expression of human high-mobility group A2 (HMGA2) in five hepatocellular carcinoma (HCC) cell lines at the mRNA and protein level. (A) Immunoblot analysis of HMGA2 levels in nonmetastatic or low metastatic potential human HCC cell lines (PLC/PRF/5, Huh-7, HepG2 and Hep3B) and high metastatic cells (MHCC97-H); β-actin was detected as a loading control. (B) The transcript levels of HMGA2, relative to *Gapdh*, determined by qRT–PCR; error bars indicate SEM, n=3.

To establish the correlation between HMGA2 expression and HCC cell lines, five HCC cell lines were evaluated for HMGA2 mRNA expression using quantitative PCR (qPCR) and expression at protein level using western blot analysis. HMGA2 was highly expressed in MHCC97-H cells that were characterized as high metastatic potential (Fig. 1A and B). In contrast, the nonmetastatic or low metastatic potential cells almost completely lacked HMGA2 or showed decreased expression (Fig. 1A and B).

### Overexpression of HMGA2 promotes, but silencing inhibits EMT

**Figure 2:**
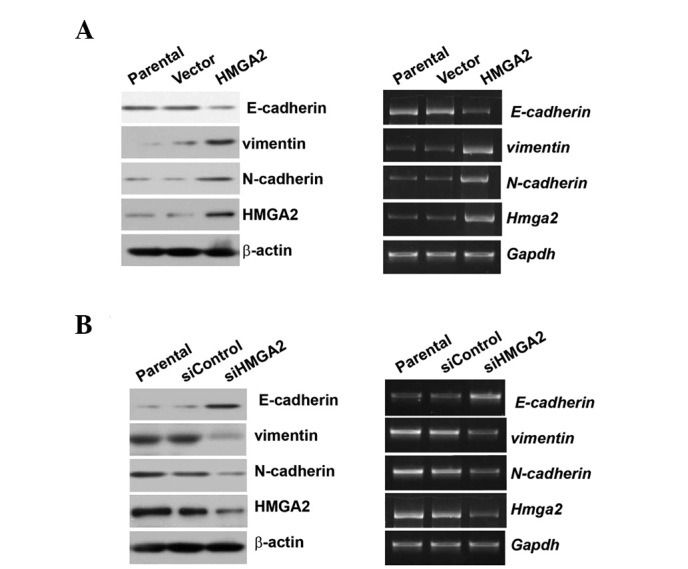
Overexpression of human high-mobility group A2 (HMGA2) promotes but silencing inhibits epithelial-to-mesenchymal transition (EMT). (A) The expression of epithelial or mesenchymal markers was detected in the overexpression of HMGA2 HepG2 cells by western blot analysis and semi-quantitative RT-PCR assay. (B) The expression of epithelial or mesenchymal markers was detected in the knockdown of HMGA2 MHCC97-H cells by western blot analysis and semi-quantitative RT-PCR.

To verify whether HMGA2 regulates EMT in HCC cell lines, HA-HMGA2 was transiently transfected into low metastatic HepG2 cells or the expression of HMGA2 was knocked down using siRNA in MHCC97-H cells. HMGA2 overexpression induced downregulation of the epithelial marker E-cadherin expression at the protein and mRNA levels; furthermore, mesenchymal genes such as vimentin or N-cadherin expression at the protein and mRNA levels were induced by HMGA2 overexpression (Fig. 2A). Whether HMGA2 knockdown also affects EMT marker variation was examined. HMGA2 knockdown could increase E-cadherin expression and decrease vimentin or N-cadherin expression at both the protein and mRNA levels (Fig. 2B).

### Ectopic expression of HMGA2 promotes, but depletion blocks tumor cell migration and invasion

**Figure 3:**
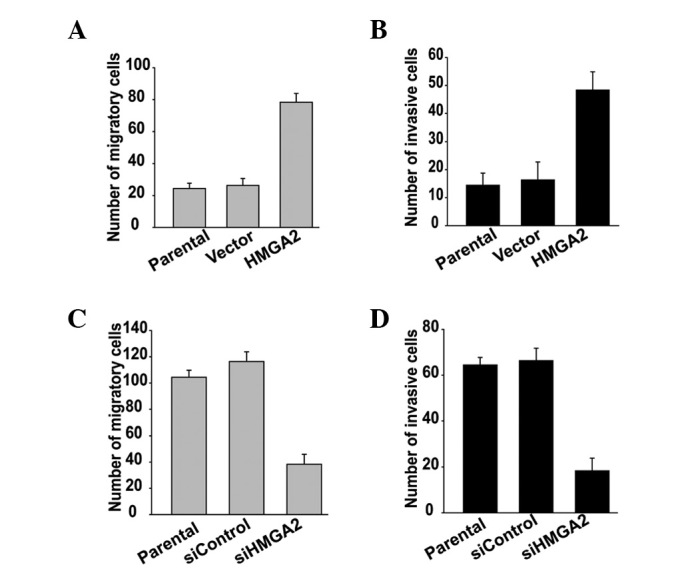
Ectopic expression of human high-mobility group A2 (HMGA2) promotes but depletion blocks tumor cell migration and invasion. (A) The overexpression of HMGA2 in HepG2 cells together with their controls were analyzed by transwell migration assay. Data are presented as mean ± SEM of 3 independent assays. (B) A transwell invasion assay was performed in overexpression of HMGA2 HepG2 cells. (C) and (D) The knockdown of HMGA2 MHCC97-H cells inhibited cell migration (C) or invasion (D).

The regulatory effect of HMGA2 on migratory and invasive ability was examined using transwell migration and invasion assays. HMGA2 overexpression promotes the migration and invasion of HepG2 cells (Fig. 3A and B), whereas knockdown of HMGA2 blocked the migration and invasion of MHCC97-H cells (Fig. 3C and D). These data suggest that HMGA2 participates in the regulation of HCC cell migration and invasion.

### HMGA2 regulates the expression of Twist and Snail at both mRNA and protein level

**Figure 4:**
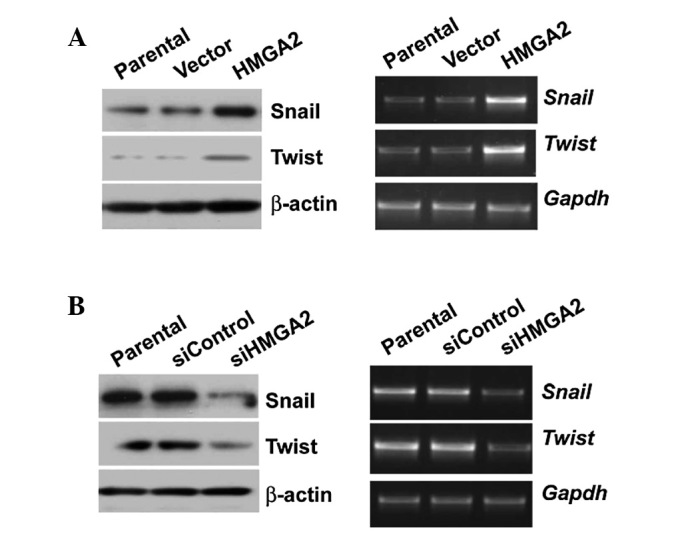
Human high-mobility group A2 (HMGA2) regulates the expression of Twist and Snail at both mRNA and protein level. (A) Overexpression of HMGA2 increased the expression of Snail and Twist in HepG2 cells. (B) Konckdown of HMGA2 decreased the expression of Snail and Twist in MHCC97-H cells.

To explore how HMGA2 promotes migration and invasion by inducing EMT at the molecular level, Twist and Snail, two strong inducers of EMT, were used. Overexpression of HMGA2 significantly upregulated Snail and Twist expression at both the mRNA and protein level (Fig. 4A). In contrast, HMGA2 knockdown decreased the expression of the two molecules (Fig. 4B).

## Discussion

*Hmga2* is a member of the HMGA family that encodes a chromatin-associated protein (3). In humans, the *Hmga2* gene is located at chromosome 12q14 and encodes a 109 amino acid protein. In embryonic tissues, HMGA2 is essential for normal cardiac development (16) and increases the frequency and self-renewal of fetal and young-adult stem cells (17), thereby regulating cell growth and differentiation. Moreover, the role of HMGA2 that participates in the process of tumorigenesis has been studied extensively. The functional significance of HMGA2 in HCC cell lines, however, is still not clear. Elucidating its functional roles and molecular mechanisms involved in the tumorigenesis may, therefore, be helpful for the early diagnosis and development of malignancies.

In this study, the expression of HMGA2 in five HCC cell lines was examined at the mRNA level using real-time PCR and at the protein level by western blot analysis. The level of HMGA2 expression among the five HCC cell lines coincided with their invasiveness, which was consistent with a previous observation that increased expression of HMGA2 correlated with increased invasiveness in tumor tissues (18). Many successive steps are required for a growing benign hyperplasia to evolve into a fully malignant and metastatic cancer (1). EMT is a critical event that enables cancer cells to invade the local tissue, acquire competence for intravasation and generate progeny with tumor-initiating capacities (2). During EMT, differentiated epithelial cells lose their cell-cell adhesions, become more motile and exhibit mesenchymal features. For example, loss of E-cadherin expression, a key molecule of the adherens junction and a tumor suppressor gene (CDH1) and induction of vimentin-based intermediate filaments are two of the many established hallmarks of the EMT process (3). To demonstrate the role of HMGA2 in EMT, the expression of HMGA2 was silenced using siRNA in MHCC97-H cells with highly invasive potential and high HMGA2 expression. Concurrently, we overexpressed HMGA2 in HepG2 cells with low invasion and HMAG2 low expression. The variation of HMGA2 expression was found to correlate significantly with the expression of several putative EMT markers. In addition, assessment of the invasive potential following transfection with HMGA2-siRNA demonstrated that the rate of cell migration was significantly reduced compared with that in siControl and mock control samples, suggesting that HMGA2 may be an important contributor to the invasion of tumor cells, and that the expression level of HMGA2 influences the metastatic behavior of HCC cells.

The large number of cellular events that characterize the mesenchymal transition are thought to be collectively regulated by a group of transcription factors that coordinate the transcriptional program of EMT. These transcriptional regulators are the zinc finger factors Snail, Snail2 (also known as Slug), ZEB1, ZEB2 and the basic helix-loop-helix factors E47 and Twist1 (Twist) (4). Previously, Moustakas and his group demonstrated that HMGA2 binds directly to the Twist or Snail promoter to induce EMT using HepG2 cells. To further confirm the conclusion and explore the molecular mechanism by which HMGA2 induces EMT, it was assumed that HMGA2 upregulates the expression of Twist and Snail in HCC cell lines. Indeed, HMGA2 was found to increase the expression of the two proteins at both the mRNA and protein level. Since it was also demonstrated that HMGA2 regulates the TGF-β signaling pathway, future research should be carried out to elucidate whether HMGA2 has correlations with TGF-β in EMT in HCC cell lines.

In conclusion, the present study is the first to show that HMGA2 effectively regulates EMT to prompt invasion and metastasis in HCC cells. The function of HMGA2 as an oncoprotein may be associated with several important molecules involved in invasion and metastasis of cancer cells. These results further indicate that HMGA2 may serve as a potential target for the development of therapies for HCC, although additional detailed studies *in vivo* are required.
